# Deaths at Cricket

**Published:** 1911-07-15

**Authors:** 


### Deaths at Cricket.

A daily paper of July 4 mentions the death of a young man, aged thirty-five, while playing cricket. He was batting, and when running from one wicket to the other suddenly collapsed within two yards of the crease. Another similar case attracted our attention in a daily paper a few years ago. A young man, aged thirty-four, had just hit a ball for five runs (presumably a boundary hit). He had, however, run one run and had just reached the opposite wicket when he fell down, said "Cover up my head," and never spoke again.

It would be interesting, if possible, to ascertain how many cases of death in young or middle-aged men take place during exertion in the course of the year. What is the nature of these sudden deaths? Are these cases of the nature of heat-stroke or of cardiac failure due to some organic disease of the heart? It is surprising how a heart which apparently has given no indication to its owner of not being perfectly sound, may suddenly fail. In these cases there is generally disease of the cardiac muscle. There may be a gumma of the septum or of some other part of the ventricles, or patchy fibrosis, possibly due to syphilitic disease of the smaller divisions of the coronary vessels. Again, disease of the intrapericardial part of the arch of the aorta may be present (also syphilitic in nature) which leads to interference with the circulation through the orifices of the coronary arteries, and disease of the muscle wall of the left ventricle in consequence. The majority of cases of sudden death in apparently healthy young middle-aged men we believe to be due to one of the above-mentioned forms of disease. Occasionally aortic valvular disease may be present which has not given rise to symptoms. The variety of aortic valvular disease, however, which occasions death in comparatively young men is almost invariably associated with disease of the intrapericardial portion of the aorta, and the reason for the sudden death is not the valvular lesion, but the degeneration of the cardiac muscle which is present.

With regard to the question of heat-stroke, the syncopal form of heat-stroke as described in the text-books does not appear to end in death as quickly as the above-mentioned cases of cardiac failure. Yet the possible effect of heat upon the heart is of interest. The hearts of gas-stokers and blacksmiths are occasionally found to be very large, and the cause of the enlargement may be indirectly due to the hot atmosphere in which the work of these men is conducted. In other words, the enlargement may be toxic in nature, due to products of metabolism which should be passed off by the kidneys, but are retained owing to the large quantity of water passed off by the skin. Acute affection of the heart, however, occurring in heat-stroke, is probably more vasomotor in origin than due to toxæmia.

